# Super-resolution microscopy in the diagnosis of platelet granule disorders

**DOI:** 10.1080/17474086.2017.1315302

**Published:** 2017-04-13

**Authors:** Alex E. Knight, Keith Gomez, Daniel F. Cutler

**Affiliations:** a Biotechnology Group, National Physical Laboratory, Middlesex, UK; b Katherine Dormandy Haemophilia Centre and Thrombosis Unit, Royal Free London NHS Foundation Trust, London, UK; c MRC Laboratory for Molecular Cell Biology, University College London, London, UK

**Keywords:** Super-resolution microscopy, structured illumination microscopy, platelet disorders, dense granules, Hermansky-Pudlak syndrome

## Abstract

**Introduction**: Platelet granule deficiencies lead to bleeding disorders, but their specific diagnosis typically requires whole mount transmission electron microscopy, which is often not available and has a number of important limitations. We recently proposed the use of advanced forms of fluorescence microscopy – the so-called ‘super-resolution’ microscopies – as a possible solution.

**Areas covered**: In this special report, we review the diagnosis of platelet granule deficiencies, and discuss how recent developments in fluorescence microscopy may be useful in improving diagnostic approaches to these and related disorders. In particular, we conclude that super-resolution fluorescence microscopy may have advantages over transmission electron microscopy in this application.

**Expert commentary**: The value of the super-resolution microscopies has been amply demonstrated in research; however, their potential in diagnostic applications is ripe for further exploration. Hematology is a field particularly likely to benefit because of the relative simplicity of sample preparation. We anticipate that the costs of the necessary instrumentation will continue to fall rapidly, making these technologies widely accessible to clinicians.

## 1. Introduction

### 1.1. Platelet structure and function

**Figure 1.**
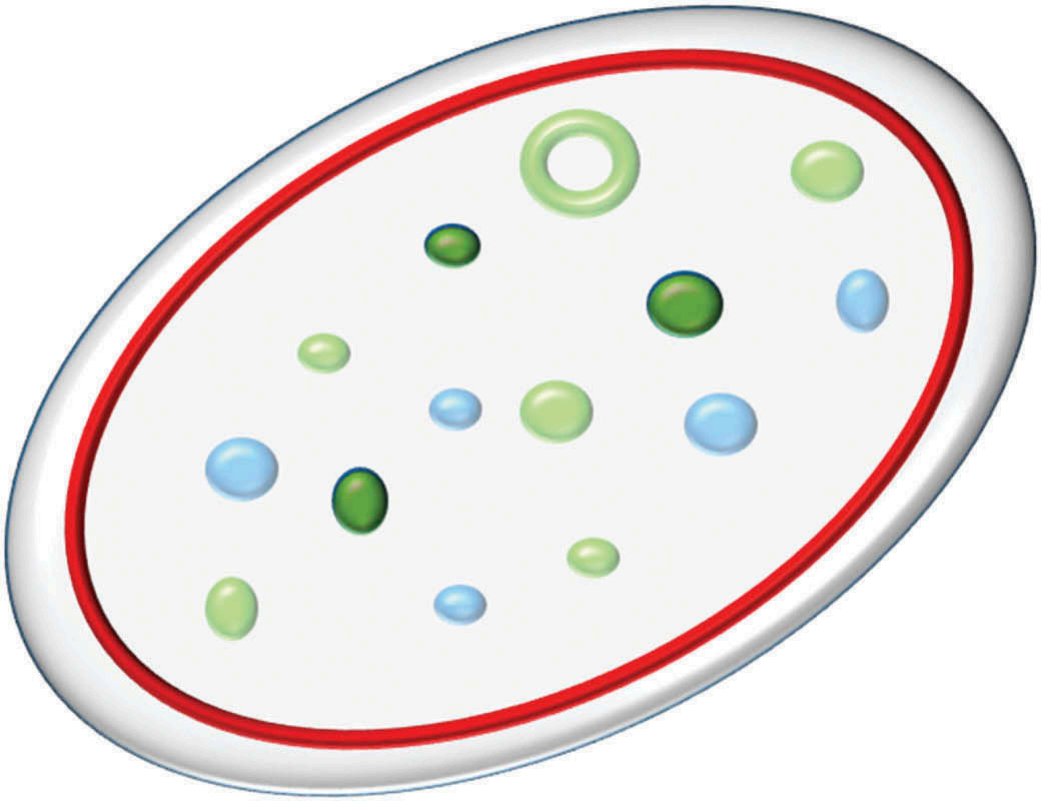
Schematic of platelet structure. A simplified schematic of the key structural features of the platelet relevant to this article. The platelet membrane, shown in pale grey, encloses internal structures including the tubulin ring (red); α-granules (pale blue); dense granules (dark green) and CD63^+^ structures (green). Note that all dense granules are CD63^+^ but that other structures are also CD63^+^; there is no unique marker for dense granules. Structures not shown for clarity include actin filaments, mitochondria, lysosomes, peroxisomes, T-granules and the canalicular system. Full color available online.

Platelets are circulating cell fragments which are best known for their critical role in hemostasis, although they perform a variety of other functions [1,2]. In humans, platelets are typically 2–5 µm in diameter and have a discoid shape due to the presence of a peripheral ring of microtubules [3]. They are produced by budding from megakaryocytes and are described as cell fragments because they do not contain a nucleus. Instead platelets contain a set of specialized organelles dedicated to their various functions (illustrated schematically in Figure 1).

These include a number of types of storage vesicles (also known as granules) whose contents are released into the plasma on platelet activation. They are traditionally divided into dense (or δ-) granules and α-granules [2]. Recent work has suggested that this binary division is overly simplistic, identifying an additional granule type [4] and apparent subdivisions within α-granules [5]. Dense granules get their name for their appearance in whole mount transmission electron micrographs; that is to say that they are electron dense (due to the presence of a high concentration of calcium).

### 1.2. Platelet granule deficiencies

A variety of platelet disorders – collectively known as storage pool disorders – are known to be associated with deficiencies in platelet granules; α-granule deficiencies in α storage pool disorder (gray platelet syndrome); dense granule deficiencies in δ-storage pool disorder, Chediak–Higashi, and Hermansky–Pudlak syndromes; and deficiencies of both types in αδ-storage pool disorder [2]. The deficiencies in these secretory granules result in a corresponding reduction in the release of their contents during platelet activation, causing an impaired clotting response.

### 1.3. Diagnosis

In many cases, to correctly diagnose these disorders, transmission electron microscopy (TEM) is required to elucidate the structural defects in the platelets but is often not readily available. Various functional assays are available, but these do not identify the structural deficit underlying a platelet disorder. Apart from the requirement for the instrumentation and associated sample preparation requirements, skilled interpretation of the images obtained is needed, as the dense granules appear on a complex background that would challenge automated image analysis [6,7]. Furthermore, the samples must be imaged on the day of preparation [8].

Vital staining with mepacrine is also possible, but this requires that the platelets be imaged on the same day. Similarly, flow cytometry approaches may be used, but also require immediate analysis, and will not reveal defects of protein localization. To improve the specificity of diagnosis for these patients, it would be useful if an alternative approach could be developed that would be more widely available.

### 1.4. Limits of resolution

An obvious alternative to TEM would be to use some form of light microscopy. In particular, fluorescence microscopy would allow staining for specific markers that would enable structures of interest within the platelet to be visualized and counted. However, platelets are only 2–5 µm in diameter; dense granules are 150 nm and alpha granules only 200–400 nm [9]. Since the resolution of conventional light microscopy is only ~200–250 nm, platelet granules cannot be visualized with sufficient clarity to permit accurate quantification and diagnosis using such a microscope (see Box 1). A potential solution to this conundrum is the newly developed family of super-resolution microscopies, which are fluorescence microscopes that can achieve spatial resolutions 2–10-fold higher than conventional light microscopy (Box 2).

**Box 1.** What is super-resolution microscopy?

Ernst Abbe showed that the spatial resolution of an image in light microscopy has a theoretical limit – this is approximately half the wavelength of the light. For visible light, this equates to approximately 200–250 nm [10,11]. This limit is caused by the diffraction of the light as it propagates through the microscope. There is no way to stop this diffraction, so it was thought that this resolution limit – sometimes called the ‘diffraction limit’ – was a fundamental limit that could not be overcome. However, recent decades have seen the development of at least three independent ways of by-passing the limit and they are beginning to make a significant impact in cell biology and related fields of research [12] – see Box 2. The importance of these innovations was recognized by the awarding of the 2014 Nobel Prize in Chemistry to some of the field’s pioneers: Eric Betzig, Stefan W. Hell, and William E. Moerner [13,14].

**Box 2.** Super-resolution microscopy methods

There are many super-resolution techniques, which are typically variants of three main approaches. These are summarized below:
**Localization microscopies**
Although the resolutions typically obtained in light microscopy (~250 nm) are much larger than molecules, individual fluorescent molecules can still be imaged and their positions localized with much higher precisions (~10 nm). In the localization microscopies, this process is repeated many thousands of times to map out the positions of many fluorescent molecules. This information can then be used to reconstruct a super-resolution image. Localization microscopy requires some way of ‘switching’ the fluorescence of the target molecules, and the need for many cycles of switching and imaging means that this approach is relatively slow. Examples include photoactivation localization microscopy (PALM) and stochastic optical reconstruction microscopy (STORM) and their variants.
**Point spread function engineering**
The shape of a diffraction limited focal spot in a microscope is referred to as the point spread function. By manipulating this point spread function, higher resolutions can be obtained. Variants include stimulated emission depletion (STED), reversible saturable optical fluorescence transitions (RESOLFT), and conical diffraction. Resolutions as fine as 20 nm can be achieved. These methods often operate by rastering a focal point through a sample, which can limit their speed somewhat compared to methods that use wide-field illumination [15].
**Structured illumination microscopies**
By using a series of patterns of illumination, and processing the resulting images mathematically, it is possible to improve spatial resolution – typically by a factor of 2 (~120 nm). The illumination patterns are typically of lines (a sinusoidal grating) or points. Because only a few images are often needed, this can be a relatively fast method. Variants include structured illumination microscopy(SIM), multifocal SIM, ‘instant’ SIM, non-linear SIM, and others.

## 2. Super-resolution imaging of platelets

### 2.1. Motivation

**Figure 2.**
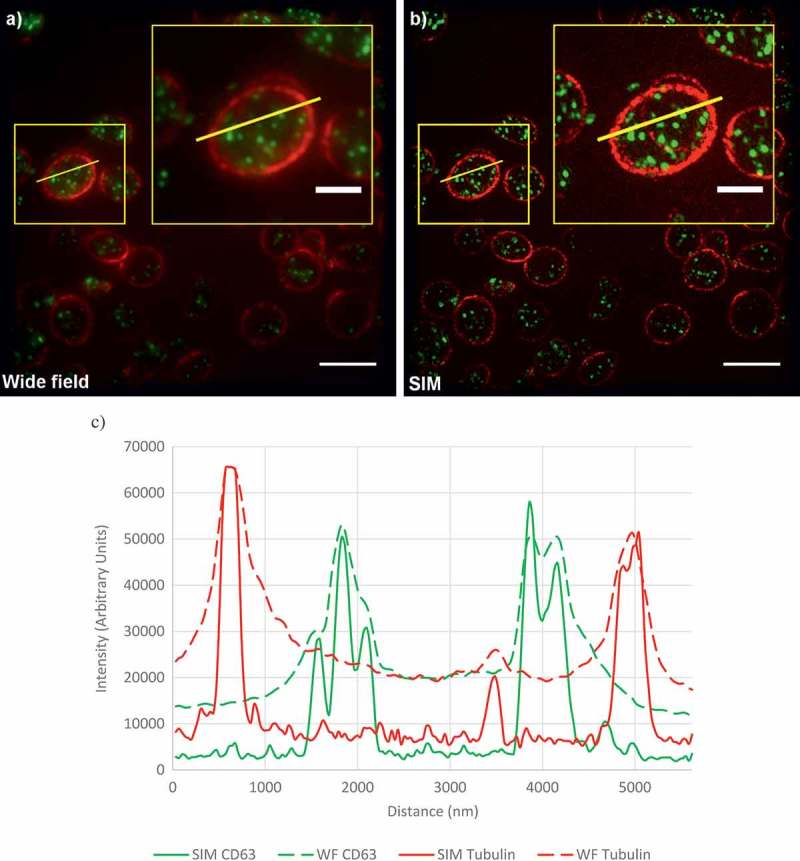
Comparison of diffraction-limited and super-resolution images of a platelet. (a) A wide-field image of platelets from a healthy control. Platelets are stained for tubulin (red) and CD63 (green). (b) A SIM reconstruction of the same field of view. Yellow boxes indicate the position of the enlarged inset image. (c) Line profiles of intensity, plotted for the yellow lines shown in (a,b). Note the much higher contrast and detail in the SIM images. All these images are calculated from the same data. Scale bars: main figures 5 µm; insets 2 µm. Full color available online.

In a recent study, we set out to demonstrate the principle of using super-resolution microscopy to diagnose platelet granule disorders [7]. From the methods listed in Box 2, we chose to evaluate the use of SIM because the spatial resolution (~120 nm) is sufficient for our purposes and the method is fast enough to be practical in a diagnostic scenario. Additionally, the sample preparation is relatively straightforward compared to the other approaches. As can be seen from the example images shown in Figure 2, SIM not only improves the resolution of the images but also the contrast, providing much clearer images that are readily suited to automated analysis.

One of the challenges we encountered is that there is currently no well-established and unique and specific protein marker for which antibodies are widely available that can be used for dense granules. We therefore chose to use as a marker the integral membrane protein CD63 which has previously been found to be correlated with dense granule deficiency [16]; however, it should be noted that this is not a unique marker for dense granules and is also localized to lysosomes. Therefore, it is important to distinguish between dense granules (defined by electron density in TEM images) and CD63+ structures (defined by fluorescence intensity in SIM images); the former are believed to be a subset of the latter.

Hermansky–Pudlak syndrome (HPS) is a well-studied disease, which in addition to dense granule deficiency is associated with a range of other symptoms including albinism that are believed to be related to deficiencies in the formation of vesicular organelles including melanosomes and lysosomes [17]. The genetics of the disease is also fairly well understood; 10 genes have been identified to date [OMIM #203300 http://omim.org/entry/203300] [18]. It therefore makes an ideal test case for this new diagnostic approach, as an unambiguous diagnosis can be made.

### 2.2. Technical approach

**Figure 3.**
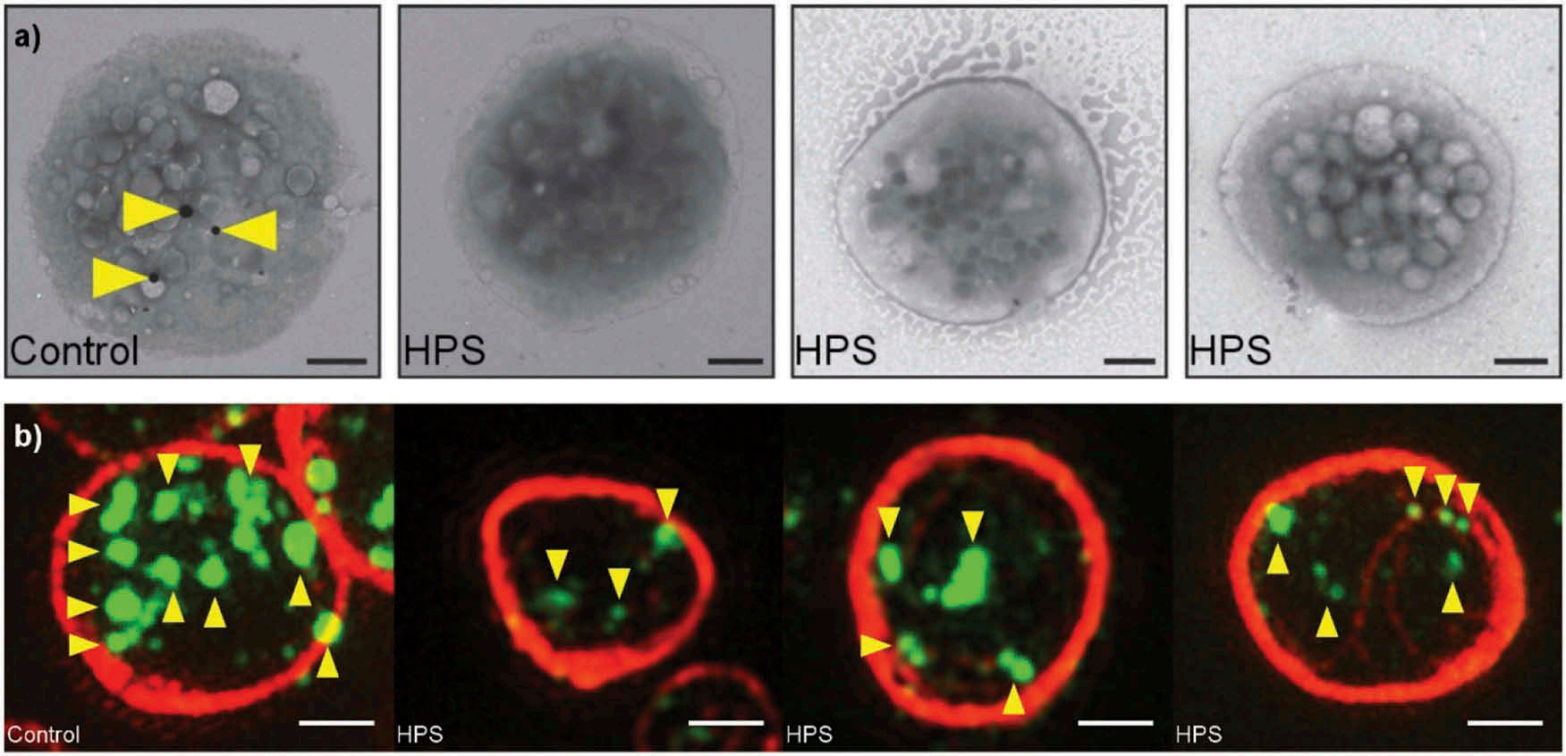
Granules in platelets from healthy controls and HPS patients. (a) Whole mount TEM images showing typical platelets from a control and three HPS patients. Dense granules are indicated with yellow arrowheads. Three are seen in the control platelet, none in the HPS platelets. (b) SIM images (typical platelets from images shown in Figure 4). Yellow arrowheads indicate the most intense CD63+ objects. The control platelet has more, and brighter, CD63+ structures. Note the variable morphology, size and intensity of the structures, which necessitates an automated algorithm to achieve consistent counting of granules. All scale bars are 1 µm. (a) Adapted from [7] under the Creative Commons Licence. Full color available online.

To evaluate the use of super-resolution microscopy in the diagnosis of this condition, we analyzed samples from seven healthy controls and three patients with different forms of HPS [7]. As a reference point, we first imaged the samples using the current gold standard whole mount TEM approach (Figure 3(a)). By TEM, we found that control platelets had an average of 3.5 dense granules, whereas very few platelets from the HPS patients contained any dense granules (average 0.07). (Please refer to the original paper [7] for further details of our methodology.)

**Figure 4.**
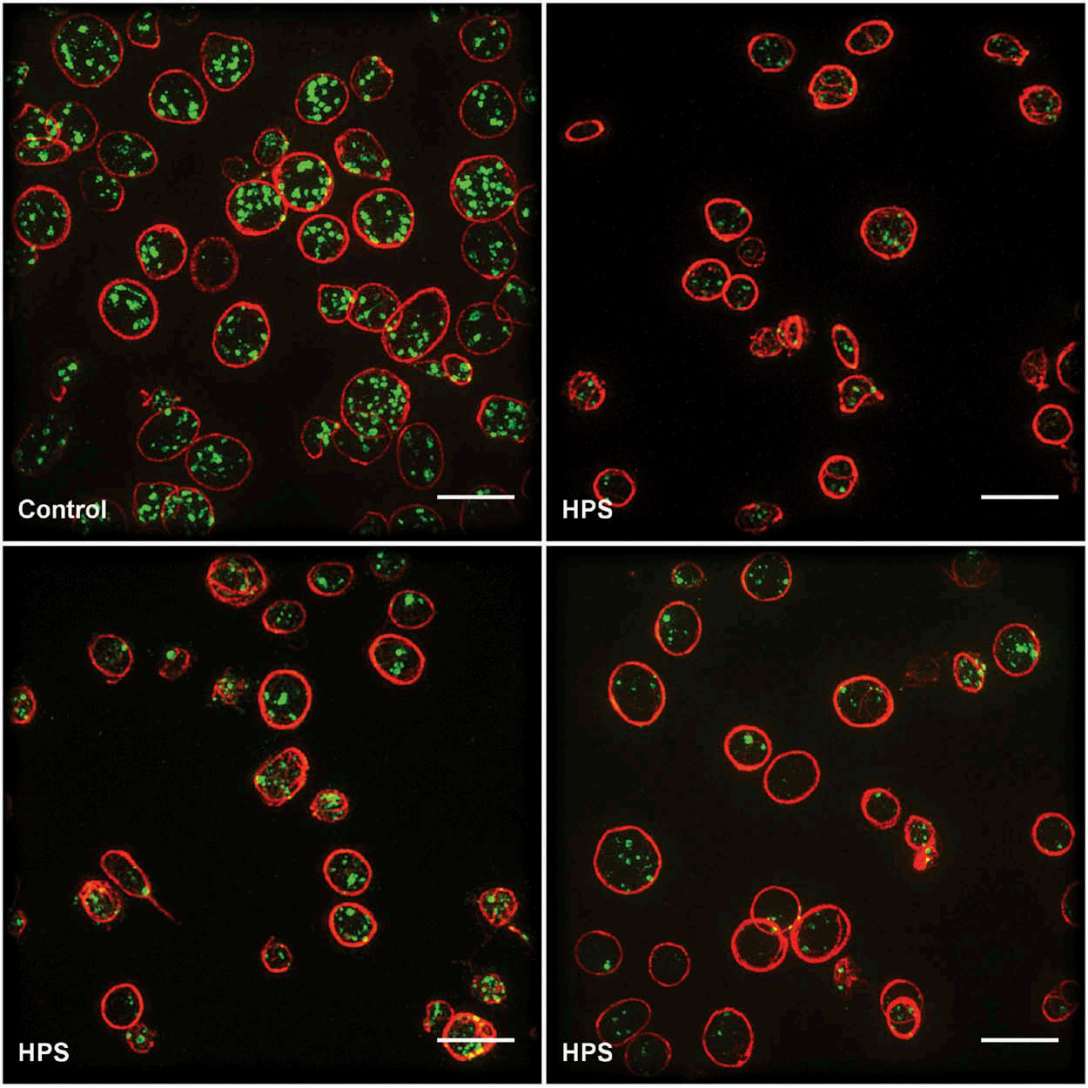
Comparison of platelets from healthy controls and HPS patients. Top left, a healthy control; top right through bottom right, HPS patients. Scale bars all 5 µm. Note the markedly more abundant and brightly stained CD63-positive granules in the platelets of the healthy control.

For SIM, the platelets were collected as platelet-rich plasma and attached to a cover slip at a moderate density to facilitate the collection of adequate statistics. The platelets were stained with antibodies to CD63 and tubulin. Visual inspection of the resulting images shows how the tubulin ring acts as a clear marker for the platelet periphery. Multiple CD63-positive organelles are clearly seen within each platelet (Figure 3(b)). Differences between the HPS and control samples are readily apparent; in the HPS patients, there are fewer CD63-positive organelles; the staining is fainter; and there appears to be additional CD63 at the platelet periphery (Figure 4).

Importantly, these impressions were confirmed by an automated image processing pipeline and statistical analysis, which we used to count the number of organelles and make comparisons between the control and HPS samples. We found that the controls had an average of 6.8 CD63-positive structures per platelet, whilst the HPS patients had only 2.4. While these numbers are higher than seen for the TEM approach, this was expected as CD63 is known not to be confined to dense granules. The use of the automated analysis meant that the control and patient samples could be distinguished with a 99% confidence level. We feel that this shows the power of the super-resolution imaging approach. The ease of differentiating patient from control, plus the potential low cost and possibly centralized facility of this approach, giving a low threshold for usage, could allow for shortening the time to definitive diagnosis for this rare disease. Furthermore, this imaging approach could be extended by the use of additional markers to create a detailed profile of platelet structures that could be used for diagnosis of a wide range of platelet disorders. For example, by adding a marker for α-granules, it would be possible to identify a range of disorders including gray platelet syndrome and αδ-storage pool disorder. However, while the published research has established proof of principle, it is clear that more research is needed to validate its clinical application. In particular, more information is needed on the variation of granule counts within healthy subjects, and on the granule counts typical of a range of granule deficiencies, to establish the wider utility of this approach.

### 2.3. Other approaches

A recent paper used a different super-resolution technique, STED (see Box 2), to image the distribution of a number of proteins which are all believed to be located in the α-granules [5]. STED has a higher resolution than SIM (as fine as ~20 nm compared to ~120 nm) but is typically somewhat slower to acquire an image [15]. Interestingly, with the higher resolution microscopy, it was shown that these proteins had different distributions, suggesting that these proteins were localized to sub-compartments within the α-granules. This reinforces our view that the detailed structural information obtained by super-resolution microscopy is an important tool for understanding platelet function and potentially for diagnosing a variety of disease-associated states of the platelet. In particular, the markers studied show the potential for investigating aspects of platelet function beyond clotting.

## 3. Future prospects

We believe that the papers described above show that super-resolution microscopy has some unique advantages as a method for investigating platelet structure and function, and potentially diagnosing structural platelet disorders. Interestingly, another recent paper described the use of super-resolution microscopy in the diagnosis of kidney disorders [19]. As with the diagnosis of platelet granule deficiencies, TEM is normally required to diagnose nephrotic disease, where the structure of the podocyte cells of the glomerulus may be disturbed. However, here the process is even more challenging, requiring a kidney biopsy and embedding and thin sectioning of the sample. Pullman *et al*. show that SIM is valuable here too, with less extensive sample preparation requirements giving a faster result and also the ability to image at high resolution in 3D. Another recent paper [20] demonstrated the use of SIM in the diagnosis of kidney cancer. Taken together with the work on platelets described above, it seems likely that there is a niche of applications in disease diagnosis and pathology where super-resolution microscopy can replace TEM, potentially reducing costs and increasing the level of detailed information available to clinicians.

Of course it is true that the first commercial super-resolution instruments were rather expensive (in the £1M plus price bracket) – but costs are likely to continue to come down rapidly, and we anticipate the introduction of a new generation of low-cost super-resolution devices. SIM is a technology which seems particularly suited to diagnostic applications because of its relatively high throughput, its ease of use, simple sample preparation, and a lack of technical obstacles to the production of relatively inexpensive instruments. Another advantage of SIM, at least for its application to platelets, is that samples can be fixed and imaged days or weeks after preparation so that not every hospital or clinic would require its own instrument.

Other advantages of SIM over TEM include the facility with which multiple markers can be imaged in parallel, the ability to image structures in 3D at high resolution without sectioning, and the ease of automating the analysis of immunofluorescent images.

For platelet disease in particular, it is likely that genetic testing will be increasingly used in diagnosis of hereditary platelet disorders. However, as new loci are still being discovered [18] and the effects of mutations vary between individuals, determining phenotype as well as genotype will continue to be important for the foreseeable future.

## 4. Expert commentary

While super-resolution microscopy has rapidly achieved awareness and increasing adoption in the cell biology and neurobiology research communities, and in related fields, there is as yet little awareness of these methodologies and their potential applications among clinicians. We feel that their potential is clear, but much more work needs to be undertaken to demonstrate their utility in the clinic. In particular, those niche areas where a subcellular structural detail is invaluable for phenotyping and potentially for diagnosis and/or stratification should be more fully explored. Different super-resolution techniques will be applicable in different scenarios. Here, we adopted SIM because although it does not give the highest spatial resolution, it is adequate to quantify platelet granules and rapidly acquired high-contrast fluorescence images. Blood cells are a particularly attractive target for super-resolution imaging because they are relatively easily obtained and sample preparation is more straightforward than other tissue types.

## 5. Five-year view

Although super-resolution microscopes are currently expensive, we anticipate that lower cost systems will soon become widely available. This will enable applications beyond pure research, including their use in the diagnosis of some types of disease. The rich information that super-resolution methods can provide on disease phenotypes at a subcellular level will complement the increasing availability of genomic data for patients.

## Key issues

- There are about 3,600 patients registered in the UK with an inheritable platelet dysfunction, representing about 14% of all registered patients with a bleeding disorder. The rarity of these disorders leads to slow initial diagnosis.
- Structural platelet disorders often involve deficiencies in certain types of granule (organelle) within the platelet.
- Because platelets are typically 2 µm to 5 µm in diameter, and the dense granules are approximately 150 nm, conventional light microscopy may not accurately measure the number of organelles within a platelet. Therefore transmission electron microscopy has been required to be able to image them.
- Structured Illumination Microscopy can resolve these structures. Although its resolution is lower than TEM, it is sufficient to accurately count granules and has the potential to be extended to observe multiple markers simultaneously, and therefore enabling the diagnosis of multiple types of granule deficiency.
- The high contrast, and the presence within the platelet of the distinctive ring of microtubules, facilitates the automation of the counting process, giving very statistically robust discrimination between patients and controls. This would be much more challenging with TEM images.
- The automated counting enables the granule counts to be obtained rapidly from many platelets per patient. This gives robust statistics which can give high confidence in diagnosing platelet disorders.
- The images contain rich information on platelet structure, beyond just the granule counts, which may be helpful in understanding platelet function and disease.
- Hermansky Pudlak Syndrome provides a good demonstration of the strengths of this approach, but this work needs to be extended to other platelet disorders – in particular those that are harder to diagnose and discriminate. To be truly comprehensive, this will acquire the use of additional markers and multi-colour imaging.
- SIM may be useful in the diagnosis of other disorders, and might represent a new platform for diagnosis in a certain subset of disorders. In particular, it also shows promise in the diagnosis of kidney disorders.
- With instrumentation costs likely to reduce, and the breadth and flexibility of application, this technology has the potential to become widely adopted in the next 5–10 years.
